# Editorial: Metabolic hormones and inflammation

**DOI:** 10.3389/fcvm.2022.1102900

**Published:** 2022-12-16

**Authors:** Matthew C. Gage, Fawaz Alzaid, Alison Delamere McNeilly, Edward A. Fisher

**Affiliations:** ^1^Department of Comparative Biomedical Sciences, Royal Veterinary College, London, United Kingdom; ^2^Dasman Diabetes Institute, Kuwait, Kuwait; ^3^INSERM UMR-S1151, CNRS UMR-S8253, Université Paris Cité, Institut Necker Enfants Malades, Paris, France; ^4^Systems Medicine, University of Dundee, Dundee, United Kingdom; ^5^Grossman School of Medicine, New York University, New York, NY, United States

**Keywords:** metabolism, inflammation, hormone, insulin, estrogen, testosterone, NPY, endothelium

The field of endocrinology impacts many aspects of physiology and pathophysiology including cardiometabolic diseases. Excitingly, new roles of hormones are continually being recognized which in addition to advancing our fundamental knowledge of our own biology, may also lead to new therapeutic strategies to be designed to combat these diseases.

In this Research Topic we have aimed to highlight new roles that metabolic hormones and their targets play in cardiometabolic diseases.

The summary schematic Figure 1 illustrates the main focus of the articles contributed. Zheng et al. discuss the evidence of how the hormone NPY (historically associated with appetite), may be a major mediator in nicotine induced endothelial dysfunction and atherosclerosis. This is important because currently, while it has been shown that nicotine exposure leads to changes in expression levels of NPY, and that the NPY system is associated with atherosclerotic cardiovascular disease, the correlation between NPY and nicotine exposure associated endothelial dysfunction are unknown.

**Figure 1:**
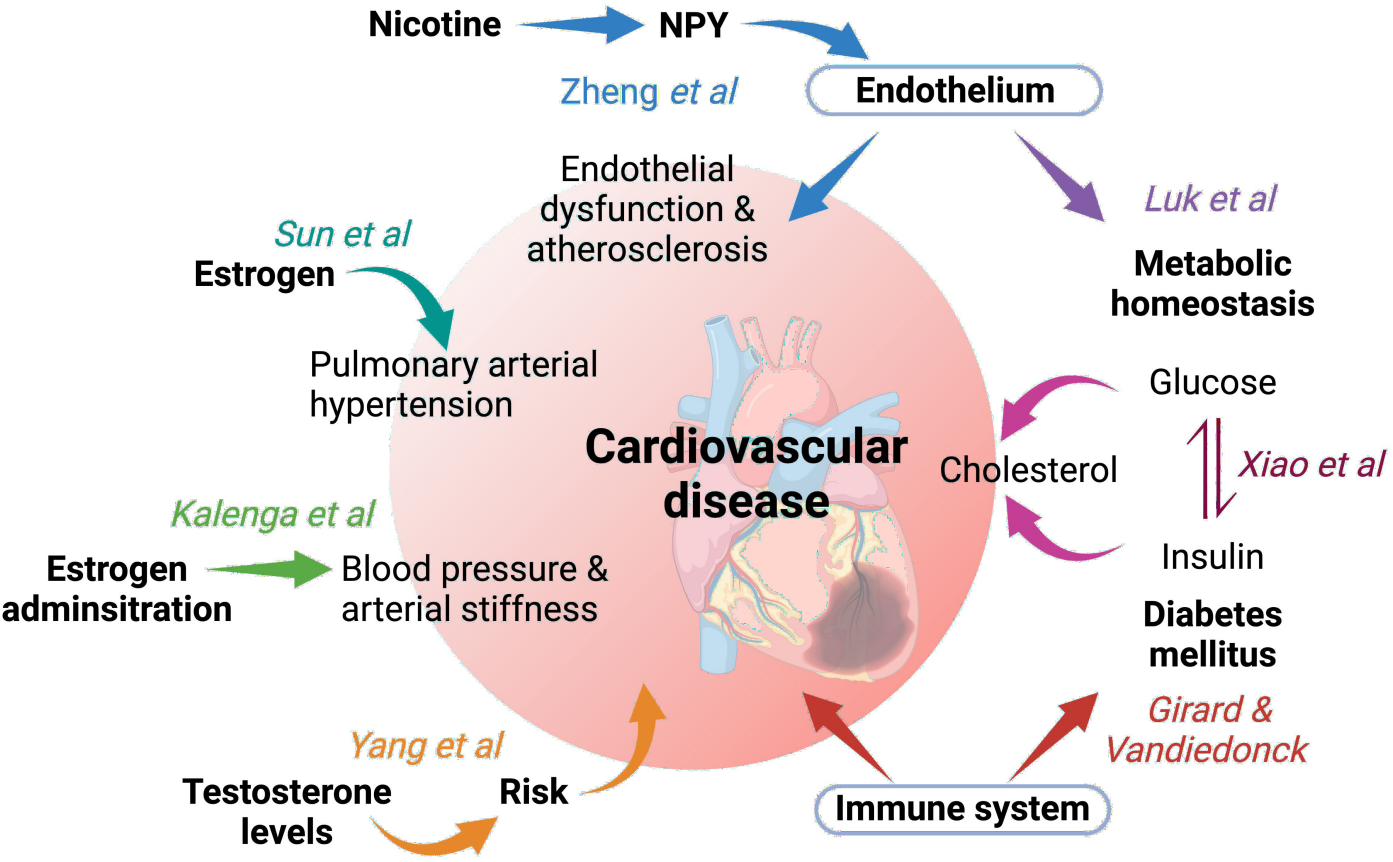
Schematic summarizing the subjects covered by the articles which have contributed to this Frontiers in Cardiovascular Medicine Research Topic “Metabolic hormones and inflammation”. Created with BioRender.com.

Keeping with the theme of the endothelium, it is the expanding roles of the endothelium in metabolic homeostasis that are the focus of Luk et al. It is now well-established that the endothelium is a key organ in mediating whole body homeostasis rather than the traditional view of being a simple barrier between the circulation and more metabolically active tissues. Adipose tissue is a key metabolic tissue—dysfunction of which is central to the metabolic complications of obesity. In this review article, Luk et al. focus on the endothelial-derived factors that are involved in the cross talk between the endothelium and adipose tissue in both a normal physiological and pathophysiological state.

Moving along from the roles of the endothelium in cardiovascular disease and metabolic homeostasis, Girard and Vandiedonck review the emerging roles of the immune system in cardiovascular disease and diabetes mellitus discussing how these diseases are increasingly considered as both inflammatory as well as metabolic. With a focus on risk-prediction, prognostics and therapeutic options under development in both type-1 and type-2 diabetes, this review highlights advances in targeting immune-related mechanisms that increase risk of cardiovascular disease. A thorough description of different leukocyte populations that contribute, by different mechanisms, and insight into systems, GWAS and post-GWAS genetic approaches that can stratify individual risk, Girard and Vandiedonck discuss the latest developments in the field.

Xiao et al. have written an important update on how glycemic control and cholesterol metabolism influence each other. Intriguingly, they discuss how genetic factors and lipid lowering drugs (such as statins and PCSK9 inhibitors) can alter glucose metabolism. Xiao et al. covers the basic mechanisms by which glucose levels influence lipid homeostasis at the transcriptional and cellular level. They then describe how cholesterol lowering drugs influence function of insulin producing and major insulin sensitive organs and tissues. Lastly, they highlight important gene variants (Hmgcr, Npc1l1, and Pcsk9), their associations with and potential roles in new-onset diabetes.

Returning to how hormones may directly affect cardiovascular diseases, Sun et al. discuss the effects of estrogen on pulmonary hypertension including important insights into the “estrogen paradox” used to describe the discrepancies between observations in patients and animal models. This review draws attention to the intriguing sex difference in pulmonary hypertension risk and severity, where although female patients are at higher risk, they have higher survival rates relative to male patients. To address this sexual dimorphism Sun et al. discuss the use of animal models and target estrogen synthesis, signaling and metabolism at multiple levels, including estrogen receptors and how their activity is regulated. Genomic regulation by engaged estrogen receptors, and non-genomic signaling pathways are put into pathophysiological contexts, broken down by effects on the vascular and immune systems, with informative graphical representations.

Estrogen is also the focus of the contribution from Kalenga et al. who investigate the association of different routes of administration of estrogen therapy in postmenopausal women with blood pressure and arterial stiffness. The global population of postmenopausal women is predicted to exceed more than one billion by 2025 and so this is a timely observation study in which Kalenga et al. show that the use of oral estrogen administration was associated with increased systolic and diastolic blood pressure compared to non-oral estrogen administration. These findings could have major implications to understanding the potential cardiovascular benefits and risks of different routes of administration of estrogen allowing individuals to make more informed decisions regarding therapy during this important transition time.

Finally, Yang et al. investigate the relationship between testosterone levels and predicting the 10-year risk of cardiovascular disease in young Taiwanese men between 30 and 49 years of age. Through a large retrospective cohort study of 1,253 participants and utilizing the Framingham Risk Score and the Atheroclerotic Cardiovascular disease Risk Estimator, Yang et al. show that a reduction in total testosterone was associated with higher cardiovascular disease risk–this new information may provide important insights into managing cardiovascular disease risk by acknowledging the legacy effect of a reduction in testosterone levels.

Together, these original contributions demonstrate the exciting potential that hormones and their less well researched targets may play in advancing new therapeutic strategies to alleviate the burden of cardiometabolic diseases.

## Author contributions

All authors listed have made a substantial, direct, and intellectual contribution to the work and approved it for publication.

